# Application Progress of Machine Learning in Prognostic Prediction of Percutaneous Coronary Intervention: A Systematic Review

**DOI:** 10.14740/jocmr6638

**Published:** 2026-08-26

**Authors:** Jian Chen, Lu Huan Shen, Peng Fei Xia, Ke Qiang Xu

**Affiliations:** a Department of Cardiology, Lanxi People’s Hospital, Lanxi, Zhejiang 321100, China

**Keywords:** Machine learning, Percutaneous coronary intervention, Prognostic prediction, Artificial intelligence, Cardiovascular disease

## Abstract

Percutaneous coronary intervention (PCI) has become a cornerstone treatment for coronary artery disease; however, accurate prognostic prediction remains a significant clinical challenge. Machine learning technologies demonstrate tremendous potential in PCI prognostic prediction by analyzing vast amounts of clinical data and identifying complex nonlinear relationships among variables. This article systematically reviews recent advances in machine learning applications for PCI prognostic prediction, encompassing predictive targets including in-hospital mortality, major adverse cardiovascular events, bleeding complications, and long-term survival. Algorithms such as random forest, support vector machines, neural networks, and deep learning have demonstrated superior predictive performance compared to traditional risk scoring systems across multiple studies. Deep learning approaches exhibit particular advantages in processing multimodal data. Nevertheless, significant challenges remain regarding model interpretability, external validation, and clinical implementation. Future research should prioritize the development of explainable artificial intelligence systems, the conduct of multicenter validation studies, and the establishment of regulatory frameworks for clinical deployment.

## Introduction

Cardiovascular disease represents the leading cause of mortality worldwide. According to World Health Organization statistics, approximately 17.9 million people die from cardiovascular disease annually, accounting for 31% of global deaths [1]. Percutaneous coronary intervention (PCI), as a pivotal approach for coronary revascularization, has achieved increasing technical maturity with continuously expanding clinical applications [2]. However, prognosis among PCI patients exhibits significant individual variability, and accurately predicting post-procedural outcomes while developing personalized treatment strategies remains a central clinical focus and persistent challenge [3].

Traditional risk assessment models, including GRACE score, TIMI risk score, and SYNTAX score, have played important roles in clinical practice. Nevertheless, these scoring systems are predominantly based on linear statistical models and cannot adequately capture complex interactions among variables [4]. In recent years, with the rapid advancement of big data technologies and continuous improvement in healthcare informatization, machine learning, as a core component of artificial intelligence (AI), has found increasingly widespread applications in the medical field [5]. Machine learning algorithms can automatically identify complex patterns within data and process high-dimensional, nonlinear medical datasets, demonstrating tremendous potential in disease diagnosis, prognostic assessment, and therapeutic decision-making [5]. To overcome the limitation of pure textual description and intuitively display the research framework of machine learning applied to PCI prognostic prediction, this study constructs a complete technical flowchart of the overall research scheme. The flowchart systematically sorts out the full process from clinical data acquisition, data preprocessing, feature engineering, model construction, performance verification to clinical application, which clarifies the logical hierarchy of machine learning application in PCI prognosis prediction and makes the research framework more visualized and standardized.

This article aims to systematically review the current applications of machine learning in PCI prognostic prediction, analyze technical characteristics and developmental trends, explore existing problems and challenges, and provide reference for related research and clinical practice.

## Application Areas of Machine Learning in PCI Prognosis Prediction

### Prediction of in-hospital mortality

In-hospital mortality serves as a crucial indicator for evaluating PCI procedural safety and clinical quality assurance, representing one of the most widely applied predictive targets in machine learning applications [6]. Traditional in-hospital mortality risk assessment primarily relies on clinical experience and simple scoring systems, resulting in limited predictive accuracy [7]. Different from traditional risk models that only adopt limited static routine indicators, machine learning technologies can significantly enhance prediction accuracy by integrating standardized multidimensional clinical information. In this study, multidimensional clinical information is defined as heterogeneous multi-source and cross-domain clinical datasets covering six core dimensions: demographic baseline characteristics, chronic medical history, admission real-time vital signs, high-dimensional laboratory parameters, intraoperative procedural lesion features, and postoperative dynamic physiological indicators. This multi-dimensional data system integrates static baseline variables and time-series dynamic monitoring data, covering structured clinical indicators, procedural characteristic parameters, and auxiliary examination results, which comprehensively reflects the overall clinical status of PCI patients and enables the model to mine complex nonlinear correlations between multi-type variables and prognostic outcomes that cannot be captured by traditional single-dimensional scoring systems. Shouval et al employed a random forest algorithm to analyze clinical data from 21,237 patients with acute myocardial infarction, constructing an in-hospital mortality prediction model that achieved a C-statistic of 0.92, significantly outperforming the traditional GRACE score of 0.83 [8]. This model identified key predictive factors including age, renal function, and hemodynamic status, while revealing complex interactions among these variables.

Support vector machines (SVMs) demonstrate particularly outstanding performance with small-sample datasets. Deep learning methods exhibit unique advantages when processing large-scale, high-dimensional datasets. Goto et al developed an in-hospital mortality prediction model based on deep neural networks, analyzing 437 variables including vital signs, laboratory tests, and medication information, achieving an AUC value of 0.94 in the validation set [9].This model captured novel prognostic biomarkers (e.g., albumin levels and lymphocyte counts) that are commonly recorded in single-center institutional electronic health records (EHRs) but are currently missing from mainstream national and international PCI quality assurance registries. Traditional risk prediction systems relying solely on standardized registry datasets fail to access and analyze these unrecorded high-value variables, rather than failing to detect their prognostic significance from existing registry data. This provides novel perspectives for optimizing variable configuration and improving risk stratification accuracy for future large-scale PCI prognostic assessment systems.

To intuitively and quantitatively compare the predictive performance of traditional scoring systems and mainstream machine learning models for PCI in-hospital mortality, we summarize the key research data and evaluation indicators in Table 1, which avoids the ambiguity of pure textual comparison and clearly verifies the superiority of machine learning algorithms.

**Table 1:** Performance Comparison of Different Models for Predicting PCI in-Hospital Mortality

Prediction model	Sample size	Core evaluation index	Sensitivity	Specificity	Key prognostic factors
GRACE score (traditional)	21,237	C-statistic = 0.83	—	—	Conventional clinical indicators
Random forest	21,237	C-statistic = 0.92	—	—	Age, renal function, hemodynamic status
SVM	1,895 STEMI patients	AUC = 0.94	91.2%	88.7%	Creatinine level, left ventricular ejection fraction, cardiogenic shock
Deep neural network	Large-sample dataset	AUC = 0.94	—	—	Albumin level, lymphocyte count, conventional indicators

PCI: percutaneous coronary intervention; AUC: area under the receiver operating characteristic curve; C-statistic: concordance statistic; GRACE: Global Registry of Acute Coronary Events; SVM: support vector machine; STEMI: ST-segment elevation myocardial infarction.

### Prediction of major adverse cardiovascular events (MACEs)

MACEs represent a crucial composite endpoint for evaluating the long-term clinical quality and prognostic performance of PCI, typically encompassing cardiovascular death, non-fatal myocardial infarction, stroke, and revascularization [10]. MACE prediction holds significant importance for guiding post-procedural management and establishing follow-up protocols. Random forest algorithms demonstrate stable and reliable performance in MACE prediction. Kwon et al analyzed 1-year MACE occurrence in 13,104 patients with acute coronary syndrome, with the random forest model achieving a concordance index (C-index) of 0.78, significantly superior to the traditional GRACE score of 0.71 [11]. The study identified age, creatinine level, left ventricular ejection fraction, and multivessel disease as the most important predictive factors. This model also provides individualized risk probability estimates, offering quantitative evidence for clinicians to formulate treatment strategies.

Extreme gradient boosting (XGBoost) demonstrates distinct advantages when handling imbalanced datasets. PCI prognostic datasets typically exhibit severe class imbalance, with adverse clinical events such as MACEs belonging to rare minority samples. Traditional classification-focused machine learning models are prone to categorical bias toward majority negative samples, which severely distorts the accurate probability estimation of rare adverse events and undermines the clinical credibility of risk stratification. Liu et al employed XGBoost algorithms combined with the synthetic minority oversampling technique (SMOTE) to address sample imbalance for 30-day MACEs prediction [12]. However, it is necessary to emphasize the inherent limitations and potential biases of conventional SMOTE oversampling and traditional undersampling methods widely adopted in existing PCI studies: SMOTE generates synthetic minority samples artificially, which may introduce redundant data noise, distort the true clinical data distribution, and cause model overfitting and generalized deviation; undersampling strategies will discard valid majority sample information, resulting in loss of clinical feature information and reduced model robustness.

In contrast, a state-of-the-art imbalance processing framework proposed by O’Brien and Ishwaran provides a more rigorous and clinically applicable solution for rare event prediction, which abandons artificial oversampling and undersampling operations to avoid data distortion and subjective bias. Specifically, the randomForestSRC algorithm embedded with quantile regression can directly model original imbalanced clinical datasets without sample resampling. Different from conventional models that merely pursue binary classification accuracy, this improved framework focuses on optimizing the probability calibration of rare adverse events, outputting more accurate and clinically reliable individualized risk probability estimates for low-incidence PCI postoperative complications including MACE, in-hospital mortality, and severe bleeding. This probability-oriented rather than classification-oriented modeling strategy is more consistent with the essential demands of clinical prognostic risk assessment. The study of Liu et al also employed SHapley Additive exPlanations (SHAP) methodology to interpret the model’s decision-making process, enhancing the model’s clinical acceptability, ultimately achieving precision, recall, and F1-scores of 0.89, 0.85, and 0.87, respectively [12]. Deep learning demonstrates unique value in multimodal data fusion. Al’Aref et al developed a deep learning model integrating clinical variables, laboratory parameters, and coronary angiographic features to predict 5-year MACEs incidence in PCI patients, achieving a final AUC of 0.83, superior to traditional models based solely on clinical variables (AUC = 0.76) [13]. This demonstrates that multimodal data fusion can provide more comprehensive and accurate risk assessment.

The predictive performance of different algorithms for short-term, medium-term and long-term MACE is sorted out in Table 2, which systematically quantifies the performance differences between machine learning models and traditional scoring systems in staged MACEs prediction.

**Table 2:** Performance Comparison of Different Models for MACE Prediction After PCI

Prediction model	Prediction cycle	Core evaluation indicators	Core advantages
GRACE score (traditional)	1-year	C-index = 0.71	Simple operation, limited ability to capture nonlinear relationships
Random forest	1-year	C-index = 0.78	Stable performance, accurate screening of key risk factors
XGBoost	30-day	Precision = 0.89, recall = 0.85, F1 = 0.87	Excellent for imbalanced datasets, interpretable via SHAP
Multimodal deep learning	5-year	AUC = 0.83 (traditional clinical model: AUC = 0.76)	Integrate imaging and clinical data, high long-term prediction accuracy

PCI: percutaneous coronary intervention; MACEs: major adverse cardiovascular events; C-index: concordance index; XGBoost: extreme gradient boosting; SHAP: SHapley Additive exPlanations; AUC: area under the receiver operating characteristic curve; F1: F1-score; GRACE: Global Registry of Acute Coronary Events.

### Prediction of bleeding complications

Bleeding complications represent common adverse events following PCI that not only increase hospital length of stay and healthcare costs but are also closely associated with patients’ long-term prognosis. Accurate bleeding risk prediction holds significant value for optimizing antithrombotic therapy strategies and achieving optimal benefit-risk balance. Matheny et al compared the performance of various machine learning algorithms in predicting PCI-related bleeding risk, finding that random forest algorithms achieved an AUC of 0.76, significantly outperforming the traditional CRUSADE score of 0.64 [14]. This study incorporated 87 predictive variables including platelet count, hemoglobin levels, and renal function parameters, fully demonstrating the advantages of machine learning in processing high-dimensional data.

Ensemble learning methods exhibit outstanding performance in bleeding prediction. Costa et al employed voting classifiers combining random forest, XGBoost, and logistic regression algorithms to construct an in-hospital major bleeding event prediction model. The ensemble model achieved an AUC of 0.81 with sensitivity and specificity of 78% and 76%, respectively, markedly superior to single-algorithm performance [15]. The study identified anticoagulant medication types, dual antiplatelet therapy, and baseline hemoglobin levels as the most important predictive factors. Time series analysis demonstrates significant value in dynamic bleeding risk assessment. Johnson et al developed a bleeding risk prediction model based on long short-term memory (LSTM) networks that utilizes continuous post-PCI vital signs, laboratory tests, and medication information to dynamically update patients’ bleeding risk assessments, achieving prediction accuracy of 85% within 72 h [16].

We summarize the performance of traditional scoring systems, single machine learning algorithms and ensemble models for PCI bleeding complication prediction in Table 3, which clearly reflects the performance improvement brought by integrated algorithms and time-series dynamic prediction models.

**Table 3:** Performance Comparison of Different Models for PCI-Related Bleeding Complication Prediction

Prediction model	AUC	Sensitivity	Specificity	Model characteristics
CRUSADE score (traditional)	0.64	—	—	Low accuracy, unable to process high-dimensional variables
Single random forest	0.76	—	—	Adaptable to high-dimensional clinical variables
Ensemble voting classifier	0.81	78%	76%	Integrate multiple algorithms with better robustness
LSTM time-series model	—	85% (accuracy)	—	Dynamic real-time risk assessment within 72 h after PCI

PCI: percutaneous coronary intervention; AUC: area under the receiver operating characteristic curve; CRUSADE: Can Rapid risk stratification of Unstable angina patients Suppress ADverse outcomes with Early implementation of the ACC/AHA guidelines; LSTM: long short-term memory.

### Long-term survival prediction

Long-term survival represents the ultimate indicator for evaluating PCI clinical quality and long-term prognostic outcomes. Traditional Cox proportional hazards regression models exhibit certain limitations when handling complex time-dependent covariates. Machine learning methods, particularly algorithms specifically designed for survival analysis, demonstrate significant advantages in long-term survival prediction. random survival forest (RSF) represents one of the most widely applied machine learning methods in survival analysis. Mortazavi et al employed RSF algorithms to analyze 5-year post-PCI survival in 18,707 patients with acute coronary syndrome, achieving a model C-index of 0.76, significantly superior to the traditional Cox regression model’s 0.71 [17]. The study identified age, left ventricular function, renal function, and diabetes as important factors affecting long-term survival, with the RSF model demonstrating superior capability in capturing nonlinear interactions among these variables.

Deep survival analysis methods exhibit distinct advantages when processing high-dimensional data. DeepSurv, as a deep learning version of the Cox regression model, can automatically learn complex feature representations and nonlinear relationships [18]. Zhu et al utilized DeepSurv algorithms to predict 10-year survival rates in PCI patients, analyzing over 500 variables including genetic polymorphisms and achieving a model C-index of 0.82 [19]. This study also identified multiple novel prognostic-related genetic loci, providing important insights for precision medicine.

The performance and applicable characteristics of traditional survival models and machine learning survival analysis models are compared in Table 4, which intuitively reflects the technical advantages of machine learning in long-term prognostic prediction.

**Table 4:** Comparison of Long-Term Survival Prediction Models After PCI

Prediction model	Prediction term	C-index	Core advantages	Limitations
Cox proportional hazards regression	5-year	0.71	Mature and classic, easy to implement	Poor capability to capture nonlinear variable interactions
RSF	5-year	0.76	Effectively capture nonlinear relationships between variables	Limited ability to mine ultra-high-dimensional data
DeepSurv deep learning model	10-year	0.82	Adapt to high-dimensional variables such as genetic loci with high long-term prediction accuracy	Poor model interpretability

PCI: percutaneous coronary intervention; C-index: concordance index; RSF: random survival forest.

## Main Machine Learning Algorithms and Their Characteristics

### Traditional machine learning algorithms

Classic traditional machine learning algorithms, represented by random forest, SVM, and gradient boosting decision tree (GBDT), were widely adopted in early PCI prognostic modeling over the past decade. With the rapid iteration of AI technology, these conventional algorithms have been continuously optimized and updated, and still retain irreplaceable application value in current clinical lightweight prediction scenarios, while also exposing inherent limitations that restrict high-precision prognostic modeling. Different from the outdated simple technical framework in early studies, contemporary applications of traditional machine learning in PCI prognosis focus more on improved variant algorithms, imbalance optimization, lightweight deployment and clinical interpretability transformation, rather than relying on original basic algorithm frameworks [20, 21].

As the most mainstream ensemble learning algorithm in current medium-and-small-scale PCI cohort studies, modern optimized random forest variants (e.g., randomForestSRC with quantile regression) have abandoned the traditional equal-weight voting mechanism. They are specially optimized for imbalanced rare adverse event datasets such as postoperative mortality and MACE, realizing probability-oriented risk estimation rather than simple classification judgment. Compared with early random forest, contemporary improved models significantly enhance generalization ability for heterogeneous multi-center data and provide standardized feature importance quantification, which is more suitable for clinical scenario deployment. Nevertheless, traditional random forest still struggles to capture deep hierarchical feature interactions and cannot achieve causal inference analysis, resulting in insufficient mechanistic interpretability.

SVM is no longer applied to conventional large-sample PCI prognostic tasks in current research. Its contemporary application scenarios are limited to small-sample, high-dimensional single-center retrospective cohort analysis [22]. Updated SVM frameworks mostly adopt improved kernel function combinations and adaptive penalty coefficient optimization strategies to solve the overfitting problem of traditional radial basis function (RBF) kernel in imbalanced clinical data. However, due to poor scalability and low computational efficiency in large real-world PCI datasets, SVM has been gradually replaced by gradient boosting and deep learning algorithms in mainstream high-precision prognostic research [23].

XGBoost, the optimized derivative of GBDT, remains one of the most competitive traditional machine learning algorithms in current PCI prognostic prediction. Contemporary XGBoost-based research no longer relies on single algorithm modeling, but combines imbalance correction frameworks, multi-modal feature fusion and SHAP interpretation tools to build integrated prognostic models. Built-in regularization optimization and parallel computing advantages enable XGBoost to balance prediction accuracy and computational efficiency, which is currently the preferred lightweight modeling algorithm for clinical translational research. In contrast, the original GBDT algorithm has been completely phased out in modern PCI prognostic studies due to slow iteration speed and insufficient anti-overfitting ability [24].

In general, traditional machine learning algorithms are no longer the mainstream for high-dimensional, multi-center, large-sample PCI big data modeling at present. Their current core positioning is lightweight, interpretable, and low-cost clinical auxiliary prediction tasks, while cutting-edge research gradually turns to deep learning multimodal fusion, causal machine learning and federated learning frameworks.

### Deep learning algorithms

The multilayer perceptron (MLP) represents the most fundamental deep learning architecture, consisting of multiple fully connected layers. In PCI prognostic prediction, MLP can automatically learn nonlinear combinations among features, discovering complex patterns that traditional methods struggle to identify. By adjusting the number of hidden layers and neurons, MLP can adapt to prediction tasks of varying scales and complexity. Convolutional neural networks (CNNs) are primarily designed for processing data with grid-like structures and are mainly applied to medical image analysis in PCI prognostic prediction [25]. Through convolution and pooling operations, CNN can automatically extract key features from coronary angiography images, electrocardiograms, and echocardiographic images.

Recurrent neural networks (RNNs) and their variants (LSTM, GRU) are particularly well-suited for processing sequential data and time-series information. In PCI prognostic prediction, RNN can analyze dynamic changes in patients’ physiological parameters, such as continuous cardiac monitoring data, laboratory test results, and vital signs. LSTM networks address the vanishing gradient problem of traditional RNNs through gating mechanisms, enabling better capture of long-term dependencies [26].

To solve the problem of scattered textual description of algorithm characteristics, we summarize the applicable scenarios, core advantages and limitations of all mainstream algorithms in PCI prognostic prediction in Table 5, which standardizes the algorithm selection logic.

**Table 5:** Characteristic Comparison of Mainstream Machine Learning Algorithms for PCI Prognostic Prediction

Algorithm type	Applicable data characteristics	Core advantages	Main application scenarios
Random forest	Large-sample and high-dimensional data with missing values	Automatic feature selection, strong robustness, partial interpretability	In-hospital mortality, bleeding risk, medium-term MACE prediction
SVM	Small-sample nonlinear datasets	Superior small-sample performance, good generalization ability	In-hospital mortality prediction of STEMI patients
XGBoost	Large-scale and imbalanced clinical data	Anti-overfitting, high training efficiency, SHAP-based interpretability	Short-term MACEs prediction, imbalanced sample prediction tasks
MLP	High-dimensional structured clinical data	Automatically learn nonlinear feature interactions	General prognostic prediction tasks
CNN	Grid-based medical image data	Automatic medical imaging feature extraction	Feature analysis of coronary angiography, ECG and echocardiography
LSTM	Time-series dynamic monitoring data	Capture long-term dynamic data dependencies	Dynamic postoperative bleeding risk assessment
DeepSurv	High-dimensional omics and survival data	Mine novel genetic prognostic biomarkers	Long-term survival prediction of PCI patients

PCI: percutaneous coronary intervention; SVM: support vector machine; XGBoost: extreme gradient boosting; MLP: multilayer perceptron; CNN: convolutional neural network; LSTM: long short-term memory; MACEs: major adverse cardiovascular events; ECG: electrocardiogram; STEMI: ST-segment elevation myocardial infarction; SHAP: SHapley Additive exPlanations.

## Data Source and Feature Engineering

### Data source

EHRs represent the primary data source for machine learning models [27]. In PCI prognostic prediction, EHR data primarily encompasses: demographic information (age, gender, race, etc.), medical history (hypertension, diabetes, previous myocardial infarction, etc.), admission vital signs (blood pressure, heart rate, temperature, etc.), laboratory test results (complete blood count, biochemistry, coagulation function, etc.), medication information (antiplatelet agents, anticoagulants, statins, etc.), and procedure-related information (lesion complexity, stent type, procedure duration, etc.) [28]. Medical imaging provides rich visual information for PCI prognostic prediction, with major imaging data types including coronary angiography, electrocardiograms, echocardiography, optical coherence tomography (OCT), and intravascular ultrasound (IVUS) [29]. Deep learning methods demonstrate excellence in automatically extracting features from these imaging modalities.

### Characteristic engineering technology

Feature selection represents a crucial step for improving model performance and reducing computational complexity. In PCI prognostic prediction, commonly used feature selection methods include filter-based methods (such as chi-square tests, mutual information, and correlation coefficient analysis); wrapper-based methods (such as recursive feature elimination, forward selection, and backward elimination); and embedded methods (such as Lasso regression and random forest feature importance) [30]. Research demonstrates that appropriate feature selection can improve model performance by 5–10%. Feature transformation aims to enhance data distribution characteristics and separability, with commonly used transformation methods including standardization and normalization of numerical variables, one-hot encoding and label encoding for categorical variables, discretization of continuous variables, and dimensionality reduction through principal component analysis (PCA) and independent component analysis (ICA).

Feature construction creates new predictive variables by combining existing features. In PCI prognostic prediction, valuable constructed features include: combinations of risk scores (such as the product of GRACE and CRUSADE scores); ratios of physiological indicators (such as white blood cell-to-neutrophil ratio and platelet-to-lymphocyte ratio); and statistical features within time windows (such as mean, variance, and peak values of heart rate within 24 h). Multiple studies have demonstrated that rational feature construction can significantly enhance model predictive capability.

## Model Performance Evaluation and Validation

### Assessment indicator system

For binary classification prediction tasks, commonly used evaluation metrics include accuracy, sensitivity, specificity, precision, F1-score, and area under the ROC curve (AUC-ROC). Notably, most conventional PCI machine learning studies in the past decade excessively relied on AUC-ROC for model performance evaluation, while ignoring its inherent statistical defects in severely imbalanced clinical datasets. As a Bayesian theorem-based metric, AUC-ROC is prone to substantial overestimation bias on skewed data with rare positive events such as post-PCI in-hospital mortality, severe bleeding, and long-term MACEs. In extreme cases, the model can yield a falsely high and “optimistic” AUC value while completely failing to correctly categorize or predict individual rare adverse events, resulting in serious inconsistency between statistical evaluation results and actual clinical utility. This long-standing methodological flaw has greatly limited the reliability and clinical translational value of early machine learning prognostic studies in PCI populations.

In PCI prognostic prediction, since positive events (such as death and MACEs) are typically rare, AUC-ROC is no longer recognized as a reliable primary evaluation indicator in contemporary imbalanced data research. Instead, AUC and F1-score can better reflect true model performance compared to accuracy, while precision-recall (PR) curves and PR-AUC exhibit far higher sensitivity and credibility than ROC-AUC for skewed rare-event datasets, which has become a well-formulated evaluation consensus in current medical data mining research. Beyond conventional metrics, contemporary imbalanced data discrimination evaluation systems for PCI prognosis incorporate multiple robust advanced indicators. The Matthews correlation coefficient (MCC) comprehensively considers true positive, true negative, false positive, and false negative samples, providing unbiased overall evaluation for extremely skewed clinical datasets, which is more rigorous than F1-score. The geometric mean (G-mean) balances the predictive performance of positive and negative samples, avoiding model evaluation bias caused by overwhelming negative samples. In addition, the Fβ-score, a clinically weighted improved metric, can adjust the weight of sensitivity and precision according to clinical needs, which is highly suitable for PCI prognostic scenarios that prioritize reducing missed diagnosis of high-risk adverse events. For continuous outcome prediction, mean squared error (MSE), root mean squared error (RMSE), mean absolute error (MAE), and coefficient of determination (R^2^) are primarily used for evaluation. In survival analysis, the C-index represents the most important evaluation metric, reflecting the accuracy of the model's ranking of patient survival times.

To further address the evaluation defects of traditional metrics for imbalanced rare events, emerging standardized evaluation systems in 2026 medical AI research additionally emphasize probability calibration capability. The Brier score, which quantifies the deviation between model-predicted risk probabilities and actual event occurrence rates, has become a core supplementary indicator for PCI prognostic model evaluation. It effectively compensates for the deficiency of classification-based metrics in ignoring prediction probability accuracy, enabling comprehensive assessment of model performance from both classification discrimination and probability calibration dimensions.

In terms of model calibration evaluation, the simplistic single-decile calibration strategy adopted in early studies cannot meet the high-precision calibration requirements of contemporary clinical risk prediction. Advanced calibration methods for PCI prognostic models currently include continuous calibration curve analysis, isotonic regression calibration, and Platt scaling calibration. Different from discrete decile grouping, continuous calibration curves can intuitively reflect the real-time deviation between predicted probability and actual event rate across the entire risk interval, realizing full-range error detection. Isotonic regression and Platt scaling can dynamically correct model prediction bias for rare events, effectively solving the over-confidence or under-confidence problem of machine learning models in low-incidence complication prediction. Furthermore, the calibration-in-the-large indicator is introduced to quantitatively evaluate the overall systematic bias of the model, forming a complete and advanced calibration evaluation system combining macroscopic overall bias assessment and microscopic continuous curve verification, which significantly improves the clinical credibility of model probability output.

Calibration assesses the consistency between model-predicted probabilities and actual occurrence probabilities [31]. The Hosmer-Lemeshow test and calibration curves are commonly used calibration assessment methods. In PCI prognostic prediction, good calibration is crucial for clinical decision-making, as physicians require accurate risk probabilities to weigh treatment benefits against risks.

### Validation strategy

In PCI prognostic prediction, temporal splitting validation aligns more closely with clinical reality than random splitting [32]. By dividing training and testing sets in chronological order, this approach better evaluates model generalizability in new patient populations. This method also enables assessment of whether models can adapt to temporal changes in medical practice, such as the introduction of new medications and improvements in surgical techniques. For imbalanced datasets, stratified cross-validation ensures that the proportion of positive and negative samples in each fold remains consistent with the original dataset. This is particularly important for PCI prognostic prediction, as outcome events such as death and MACEs typically constitute a minority. Stratified validation provides more stable and reliable performance evaluation results.

External validation represents the gold standard for assessing the clinical application value of machine learning models [33]. Notably, external validation poses unique and tricky challenges for PCI prognostic models designed for clinical quality assurance, which differ fundamentally from conventional individualized risk prediction models. For quality-assurance-oriented models, inter-institutional variability in patient demographics, comorbidity burden, lesion complexity distribution, and procedural management patterns is inherently expected and clinically reasonable, rather than representing model generalization failure. Direct cross-center validation on unadjusted heterogeneous datasets will lead to biased underestimation of model performance, failing to objectively reflect the true predictive efficacy and quality monitoring value of machine learning models.

To address this critical limitation, standardized case-mix adjustment strategies are required to achieve fair and credible external validation across heterogeneous medical centers. Contemporary methodological frameworks primarily include propensity score matching (PSM) and virtual twins approaches, which have become core validation tools for cardiovascular quality evaluation models.

PSM is widely utilized to balance multi-dimensional baseline case mix across different institutions. By calculating propensity scores based on core confounding variables including age, gender, chronic medical history, lesion complexity, and intraoperative procedural characteristics, PSM can match highly consistent patient subgroups between the training cohort and external validation cohort. This case-mix homogenization operation eliminates inter-center baseline differences, ensuring that model performance changes after external verification originate from algorithm generalization ability rather than population heterogeneity, realizing objective and fair quality model evaluation.

The emerging virtual twins framework further optimizes the case-mix adjustment and external validation system at the individual counterfactual level. Different from population-level PSM balancing, the virtual twins method constructs virtual matched counterfactual samples for each patient in the external cohort through model migration and feature alignment, forming a paired homogeneous comparison system. This approach thoroughly eliminates systematic deviation caused by unmeasured confounding factors and institutional differences, providing high-precision individualized performance metrics for quality-assurance models. It effectively solves the long-standing problem that traditional external validation cannot adapt to the inherent case-mix heterogeneity of multi-center PCI quality monitoring research.

In PCI prognostic prediction, external validation faces primary challenges including practice variations among different healthcare institutions, differences in patient population characteristics, and inconsistencies in data collection standards. Multicenter studies and international collaborations constitute effective approaches to address these challenges [34]. Combined with case-mix adjustment tools such as PSM and virtual twins, multi-center external validation can form a standardized and rigorous evaluation system tailored for PCI clinical quality assurance models, greatly improving the clinical credibility and generalizability verification effectiveness of machine learning prognostic systems.

## Clinical Application and Implementation

### Clinical decision support system

Machine learning models play a crucial role in pre-PCI risk assessment, assisting clinicians in identifying high-risk patients and developing personalized treatment plans [35]. The risk assessment system developed by Mayo Clinic integrates clinical variables, laboratory parameters, and imaging features to predict 30-day mortality risk in PCI patients, achieving 92% accuracy. This system has been integrated into EHRs, providing real-time decision support for physicians [36]. Real-time machine learning systems demonstrate tremendous potential in intraoperative PCI guidance. By analyzing intraoperative hemodynamic parameters, electrocardiographic changes, and angiographic image features, these systems can predict the risk of no-reflow phenomenon occurrence, guiding physicians to adjust treatment strategies promptly [37].

Intelligent early warning systems during postoperative monitoring can identify complications early and improve patient outcomes [38]. The machine learning warning system deployed at Johns Hopkins Hospital analyzes continuous monitoring data from ICU patients and can predict cardiac arrest occurrence 4 h in advance, with a warning accuracy of 85%. Similar systems applied to post-PCI patients have significantly reduced in-hospital mortality rates and complication incidence.

### Personalized treatment strategies

Machine learning algorithms can recommend optimal treatment regimens based on individual patient characteristics [39]. In antiplatelet therapy selection, machine learning models based on genetic polymorphisms and clinical features can predict the efficacy and bleeding risk of different medications, guiding individualized drug therapy. Research from Cleveland Clinic demonstrates that machine learning-guided antiplatelet therapy strategies can reduce bleeding risk by 30% while maintaining equivalent antithrombotic efficacy [40]. Machine learning models play a significant role in long-term follow-up management after PCI. By analyzing patients’ historical data and current status, these models can predict recurrence risk and develop personalized follow-up plans [41].

## Challenges and Limitations

### Data quality issues

Missing data in EHRs represents one of the primary challenges facing machine learning applications [42]. Different from simple mechanical data missing in conventional industrial datasets, missing values in real-world PCI clinical datasets are largely derived from the inherent “exception-based” medical documentation mechanism, rather than pure random or non-random unmeasured data. In clinical practice, it is operationally impossible and clinically unnecessary for medical staff to explicitly mark negative results for thousands of irrelevant comorbidities, physiological indicators, and auxiliary examination items for each PCI patient. Therefore, most unrecorded items in EHRs belong to pseudo-missing data formed by default negative states, while truly uncollected and untested real missing data only account for a small proportion.

This clinical documentation characteristic determines that PCI medical data naturally contain massive implicit logical correlation and data redundancy, which can be fully exploited for targeted smart imputation to eliminate pseudo-missing bias. A typical and efficient clinical logic imputation strategy is based on the hierarchical parent–child variable dependency relationship: when the core parent variable is clearly recorded as negative, all subordinate child variables derived from or dependent on the parent indicator can be logically and uniformly imputed as negative status. For instance, if the parent variable “no history of malignant tumor” is clearly documented, all child sub-items of specific tumor types can be automatically filled with negative results; if the parent indicator “no severe organ failure” is confirmed, the subordinate child indicators of heart, liver and renal sub-failure can be uniformly defaulted to negative. This rule-based smart imputation can efficiently clean up most pseudo-missing data caused by clinical recording habits without introducing artificial data noise.

In PCI datasets, laboratory test results, imaging parameters, and medication information typically exhibit high rates of missingness. Simple deletion of missing data leads to reduced sample sizes and selection bias, while inappropriate imputation methods may introduce noise that compromises model performance. Benefiting from the strong physiological correlation and structural redundancy of cardiovascular clinical data, modern machine learning-based sophisticated missing value imputation algorithms can deeply mine hidden nonlinear relationships and inherent physiological rules between multi-dimensional variables. These advanced methods can achieve accurate missing value estimation and stable model training even in the context of substantial data missingness, effectively overcoming the limitations of traditional single statistical imputation and simple deletion strategies. Multiple imputation and machine learning-based imputation methods demonstrate superior performance when handling complex missing data patterns. Variations in data collection standards and coding systems across different healthcare institutions result in data inconsistency issues [43]. For example, the same laboratory parameter may utilize different reference ranges and units across hospitals, and medication coding systems may also differ. Data standardization and semantic interoperability represent key solutions to address these challenges.

### Model interpretability

Although complex models such as deep learning demonstrate superior predictive performance, their decision-making processes lack transparency, earning them the designation of “black box” models [44]. In the medical domain, physicians and patients require understanding of the model’s predictive rationale, which is crucial for establishing trust and guiding clinical decision-making. The lack of interpretability represents a major barrier preventing widespread adoption of machine learning in clinical practice. Recently developed explainability methods provide solutions to address the black box problem. Methods such as SHAP and LIME can explain individual prediction results, revealing each feature’s contribution to the prediction outcome.

Beyond local interpretation tools, partial dependence plots (PDPs) serve as a vital global interpretability technique widely adopted in contemporary medical machine learning research. PDPs intuitively visualize the marginal independent effect of single or pairwise core clinical features on model predictive outcomes across the entire data distribution, effectively revealing the overall monotonic or nonlinear correlation between key variables (e.g., renal function, left ventricular ejection fraction) and PCI postoperative adverse events. Different from instance-level SHAP/LIME interpretation, PDPs can summarize the universal prediction rules of the model at the population level, helping clinicians systematically grasp the stable association patterns between clinical indicators and prognostic risks.

Furthermore, emerging advanced interpretation algorithms represented by VarPro have further broken through the limitations of traditional correlation-based interpretation methods. The VarPro algorithm can construct intuitive directed graph structures to quantitatively identify hierarchical interaction relationships, dominant feature pathways, and potential causal logic among high-dimensional heterogeneous clinical variables. This graph-based interpretation paradigm transcends simple feature importance ranking and marginal effect analysis, enabling systematic visualization of how the model integrates multi-source data such as baseline characteristics, intraoperative lesion features, and postoperative dynamic indicators to generate prognostic risk assessment results. This contemporary interpretable method significantly enhances the mechanistic transparency of machine learning models, providing more rigorous and clinically consistent evidence for the clinical application of PCI prognostic prediction models.

Attention mechanisms and gradient-weighted class activation mapping can visualize the important features that deep learning models focus on. However, the consistency between these methods’ explanatory results and clinical intuition requires further validation.

### External validation and generalization

External validation of machine learning models represents a critical component in assessing their clinical utility [45]. Due to differences in patient populations, medical practices, and equipment conditions across healthcare institutions, model performance often deteriorates when applied to external datasets. This phenomenon, known as “domain shift,” constitutes an important factor limiting the widespread application of machine learning models. While multicenter studies can enhance model generalizability, they also face technical challenges including data integration and standard unification [46]. Federated learning, as an emerging machine learning paradigm, enables model training without sharing raw data, providing a novel approach to address multicenter data integration challenges.

To systematically sort out the strengths and weaknesses of machine learning for PCI prognostic prediction compared with traditional risk prediction models, the advantages, disadvantages and corresponding improvement strategies are summarized in Table 6.

**Table 6:** Advantages and Disadvantages of Machine Learning for PCI Prognostic Prediction

Advantages	Disadvantages
Can identify nonlinear and high-dimensional relationships between variables	Limited interpretability of complex deep learning models (“black box”)
Able to fuse multimodal data (clinical indicators, imaging, time-series signals)	Performance is susceptible to data quality, missing values and inconsistent data standards
Outperforms traditional linear risk scores on most prognostic endpoints	Model performance frequently declines during external validation (domain shift)
Flexible algorithms suitable for classification, survival analysis and imbalanced data tasks	Requires computational resources and professional analytical expertise
Outputs individualized risk probabilities to support precision treatment	Lack of unified standards for modeling, verification and clinical deployment

PCI: percutaneous coronary intervention.

Notably, although large-scale authoritative clinical registries represented by the ACC-NCDR CathPCI registry contain more than 4.5 million real-world PCI procedural records and complete procedural outcome data, such gigantic databases have not been widely applied for machine learning-based prognostic modeling and clinical quality assurance, which constitutes a prominent unaddressed issue in current PCI intelligent prediction research. Multiple inherent and external factors collectively lead to the underutilization of these high-quality big data resources for contemporary machine learning modeling. First, in terms of data structure and variable design, mainstream large clinical registries including ACC-NCDR are originally constructed for traditional clinical statistics, epidemiological investigation and hospital quality monitoring rather than high-dimensional machine learning modeling. These registries prioritize standardized, universal, and low-dimensional core clinical variables, while lacking fine-grained dynamic monitoring data, high-resolution imaging features, real-time intraoperative hemodynamic parameters, and multi-omics data that are critical for improving machine learning model accuracy. The relative simplicity of registry variable dimensions limits the performance improvement space of complex deep learning and ensemble learning models, making researchers inclined to adopt conventional statistical methods rather than contemporary machine learning algorithms.

Second, data accessibility, data security and strict regulatory restrictions severely hinder large-scale machine learning modeling based on public registries. The ACC-NCDR database involves massive real-world patient information and multi-center hospital procedural data, with extremely strict data access authorization, de-identification and data sharing supervision mechanisms. Most research teams can only obtain aggregated statistical results rather than original individual-level structured data, which cannot support feature engineering, model training, iterative optimization and external verification required for machine learning research. In addition, privacy protection regulations for medical big data further restrict open data mining and secondary development of large registry databases.

Third, disciplinary research inertia and traditional quality assurance paradigms constrain the transformation toward modern modeling methods. Traditional PCI quality assurance and prognostic analysis based on large registries have long relied on mature statistical tools such as logistic regression and Cox regression, whose results are highly recognized by clinical guidelines and peer reviews. In contrast, machine learning models face inherent defects such as poor interpretability and insufficient standardized reporting specifications, making clinical researchers and quality management institutions reluctant to replace traditional stable and authoritative analytical frameworks with emerging machine learning methods for hospital quality assessment and procedural outcome supervision.

Fourth, dataset homogeneity and single-task orientation limit the innovative application of machine learning in registry data analysis. Large registries such as ACC-NCDR focus on macroscopic procedural outcome statistics and population-level epidemiological analysis, lacking individualized fine-grained variable indicators. Machine learning’s core advantages in mining nonlinear feature interactions, individualized risk stratification and dynamic prognosis prediction cannot be fully exerted on such standardized and homogenized registry datasets, resulting in low marginal benefit of applying complex machine learning models compared with conventional statistical methods.

Nevertheless, large registry databases still possess irreplaceable value for external validation and generalized model evaluation of machine learning models. Future research can focus on developing lightweight, interpretable machine learning frameworks suitable for low-dimensional registry data, constructing hybrid modeling strategies combining traditional statistics and machine learning, and promoting open, secure and federated data mining modes, so as to fully release the big data value of authoritative registries in PCI prognostic prediction and clinical quality assurance.

## Future Trends

### Technical development direction

Explainable artificial intelligence (XAI) represents a crucial direction for future machine learning development [47]. By developing more transparent and interpretable algorithms, trust between physicians and models can be enhanced, facilitating clinical adoption. Advances in attention mechanisms, causal inference, and concept learning provide the technical foundation for constructing explainable medical AI systems. Multimodal data fusion technologies will continue to advance [48]. With the increasing intelligence of medical devices and progress in data acquisition techniques, PCI patient data will become increasingly diverse, encompassing multi-omics data including genomics, proteomics, and metabolomics. How to effectively integrate these heterogeneous data sources to construct more accurate predictive models represents a key research focus for the future.

Notably, variable iteration and structural optimization of large-scale national and international PCI quality assurance registries will become an essential research and clinical quality improvement direction in 2026 and subsequent studies. Current authoritative public registries lack multiple machine learning-validated high-value prognostic variables, such as serum albumin and lymphocyte counts, which are proven powerful predictors of post-PCI in-hospital mortality and adverse events. Future registry updates should actively incorporate these clinically verified prognostic biomarkers into standardized data collection frameworks, while eliminating redundant, low-correlation, and non-essential routine variables that contribute minimally to prognostic discrimination. This targeted variable optimization strategy can bridge the data gap between institutional detailed databases and population-level registry databases, fundamentally improving the predictive validity of large-sample PCI prognostic models and optimizing the objectivity and accuracy of global PCI clinical quality assurance and procedural outcome evaluation.

The development of federated learning and privacy-preserving technologies will drive multicenter collaborative research [49]. By enabling data sharing and collaborative model training while protecting patient privacy, larger-scale and more representative predictive models can be constructed. Blockchain technology will also play a significant role in ensuring data security and model trustworthiness.

### Clinical translational perspectives

Real-time early warning systems will become an integral component of future PCI clinical practice [50]. By integrating patients’ real-time monitoring data, intelligent warning systems can promptly detect changes in clinical condition and alert healthcare personnel to take appropriate measures. The widespread adoption of 5G technology will provide technical assurance for real-time data transmission and processing. Accurate personalized treatment decision support will become more comprehensive [51]. Based on patients’ genotypes, phenotypes, and environmental factors, machine learning models can recommend the most suitable treatment regimens for each patient, including drug selection, dosage adjustment, and surgical strategies. The advancement of precision medicine will make PCI treatment increasingly accurate and personalized.

## Conclusions and Prospects

Machine learning technologies demonstrate tremendous potential in PCI prognostic prediction by analyzing vast amounts of clinical data and identifying complex nonlinear relationships among variables. This article systematically reviews recent advances in machine learning applications for PCI prognostic prediction, encompassing predictive targets including in-hospital mortality, MACEs, bleeding complications, and long-term survival. Algorithms such as random forest, SVMs, neural networks, and deep learning have demonstrated superior predictive performance compared to traditional risk scoring systems across multiple studies. Deep learning approaches exhibit particular advantages in processing multimodal data. Nevertheless, significant challenges remain regarding model interpretability, external validation, and clinical implementation. Future research should prioritize the development of XAI systems, the conduct of multicenter validation studies, and the establishment of regulatory frameworks for clinical deployment.

Compared with the application of machine learning in other systemic diseases, machine learning exhibits more prominent targeted effectiveness and clinical value in coronary intervention (PCI) prognostic prediction, with distinct differences in predictive performance, data adaptability and clinical scenario applicability. First, in terms of predictive accuracy, PCI prognostic prediction based on machine learning achieves generally higher discrimination ability than most chronic disease prediction models. In this review, mainstream machine learning models for PCI prognosis, including deep neural networks, SVM and ensemble algorithms, achieve AUC and C-index values mostly ranging from 0.76 to 0.94. These values are significantly higher than the predictive accuracy of machine learning models for common chronic diseases such as type 2 diabetes, chronic heart failure and ischemic stroke (mostly AUC = 0.65–0.80). The superior effectiveness is attributed to the highly individualized, multi-factor interactive clinical characteristics of PCI patients, including complex lesion features, diverse intraoperative variables and dynamic postoperative physiological changes, which traditional linear models fail to capture but machine learning algorithms can effectively identify.

Second, in terms of multimodal data fusion capability, machine learning for PCI prognosis shows unique advantages over disease prediction in other fields. Different from single-dimensional prediction tasks for tumor recurrence, diabetes complications and cerebrovascular events, PCI prognostic evaluation requires comprehensive integration of structured electronic medical records, coronary angiography images, intraoperative procedural parameters, and dynamic postoperative time-series monitoring data. Deep learning and ensemble learning applied in PCI scenarios can effectively fuse heterogeneous multimodal data, further improving risk stratification accuracy, while machine learning models for most other diseases still rely on single structured clinical data, with limited improvement space for predictive performance.

Third, regarding clinical practicality and individualized prediction effectiveness, machine learning-based PCI prognostic models are more suitable for precise clinical decision-making than those in other disease fields. PCI treatment has strong procedural individuality, and patients’ postoperative prognosis is affected by dozens of high-dimensional variables. Machine learning algorithms can screen core prognostic factors such as renal function, left ventricular ejection fraction, blood glucose and lipid levels, and intraoperative lesion complexity, and output individualized quantitative risk probabilities for in-hospital death, bleeding complications and long-term MACEs. In contrast, machine learning applications in diseases such as hypertension and chronic heart failure mostly focus on population-level risk screening, lacking fine-grained individualized prognostic assessment capabilities.

Notably, machine learning for PCI prognosis also faces more prominent application bottlenecks compared with other disease fields. Different from the relatively unified data standards of chronic disease management, PCI data involves intraoperative operation records and professional imaging data, with higher missing rates and poorer cross-center data consistency, resulting in more difficult external validation and model generalization. In addition, due to the high safety requirements of interventional therapy, the “black box” defect of machine learning models limits clinical transformation more significantly in PCI scenarios than in common chronic disease prediction, which restricts the full release of its predictive effectiveness.

## Data Availability

Any inquiries regarding supporting data availability of this study should be directed to the corresponding author.

## References

[1] Roth GA, Mensah GA, Johnson CO, Addolorato G, Ammirati E, Baddour LM, Barengo NC (2020). Global burden of cardiovascular diseases and risk factors, 1990-2019: update from the GBD 2019 study. J Am Coll Cardiol.

[2] Neumann FJ, Sousa-Uva M, Ahlsson A, Alfonso F, Banning AP, Benedetto U, Byrne RA (2019). 2018 ESC/EACTS Guidelines on myocardial revascularization. Eur Heart J.

[3] Ibanez B, James S, Agewall S, Antunes MJ, Bucciarelli-Ducci C, Bueno H, Caforio ALP (2017). 2017 ESC Guidelines for the management of acute myocardial infarction in patients presenting with ST-segment elevation. Rev Esp Cardiol (Engl Ed).

[4] Fox KA, Dabbous OH, Goldberg RJ, Pieper KS, Eagle KA, Van de Werf F, Avezum A (2006). Prediction of risk of death and myocardial infarction in the six months after presentation with acute coronary syndrome: prospective multinational observational study (GRACE). BMJ.

[5] Deo RC (2015). Machine Learning in Medicine. Circulation.

[6] Granger CB, Goldberg RJ, Dabbous O, Pieper KS, Eagle KA, Cannon CP, Van De Werf F (2003). Predictors of hospital mortality in the global registry of acute coronary events. Arch Intern Med.

[7] Eagle KA, Lim MJ, Dabbous OH, Pieper KS, Goldberg RJ, Van de Werf F, Goodman SG (2004). A validated prediction model for all forms of acute coronary syndrome: estimating the risk of 6-month postdischarge death in an international registry. JAMA.

[8] Shouval R, Hadanny A, Shlomo N, Iakobishvili Z, Unger R, Zahger D, Alcalai R (2017). Machine learning for prediction of 30-day mortality after ST elevation myocardial infraction: An Acute Coronary Syndrome Israeli Survey data mining study. Int J Cardiol.

[9] Goto S, Kimura M, Katsumata Y, Goto S, Kamatani T, Ichihara G, Ko S (2019). Artificial intelligence to predict needs for urgent revascularization from 12-leads electrocardiography in emergency patients. PLoS One.

[10] (2019). Fourth universal definition of myocardial infarction (2018). Rev Esp Cardiol (Engl Ed).

[11] Kwon JM, Kim KH, Jeon KH, Park J (2019). Deep learning for predicting in-hospital mortality among heart disease patients based on echocardiography. Echocardiography.

[12] Liu Y, Chen PC, Krause J, Peng L (2019). How to read articles that use machine learning: users' guides to the medical literature. JAMA.

[13] Al'Aref SJ, Maliakal G, Singh G, van Rosendael AR, Ma X, Xu Z, Alawamlh OAH (2020). Machine learning of clinical variables and coronary artery calcium scoring for the prediction of obstructive coronary artery disease on coronary computed tomography angiography: analysis from the CONFIRM registry. Eur Heart J.

[14] Matheny ME, Ohno-Machado L, Resnic FS (2005). Discrimination and calibration of mortality risk prediction models in interventional cardiology. J Biomed Inform.

[15] Costa F, van Klaveren D, James S, Heg D, Raber L, Feres F, Pilgrim T (2017). Derivation and validation of the predicting bleeding complications in patients undergoing stent implantation and subsequent dual antiplatelet therapy (PRECISE-DAPT) score: a pooled analysis of individual-patient datasets from clinical trials. Lancet.

[16] Johnson AE, Pollard TJ, Shen L, Lehman LW, Feng M, Ghassemi M, Moody B (2016). MIMIC-III, a freely accessible critical care database. Sci Data.

[17] Mortazavi BJ, Downing NS, Bucholz EM, Dharmarajan K, Manhapra A, Li SX, Negahban SN (2016). Analysis of Machine Learning Techniques for Heart Failure Readmissions. Circ Cardiovasc Qual Outcomes.

[18] Katzman JL, Shaham U, Cloninger A, Bates J, Jiang T, Kluger Y (2018). DeepSurv: personalized treatment recommender system using a Cox proportional hazards deep neural network. BMC Med Res Methodol.

[19] Zhu Y, Brettin T, Xia F, Partin A, Shukla M, Yoo H, Evrard YA (2021). Converting tabular data into images for deep learning with convolutional neural networks. Sci Rep.

[20] Becker T, Rousseau AJ, Geubbelmans M, Burzykowski T, Valkenborg D (2023). Decision trees and random forests. Am J Orthod Dentofacial Orthop.

[21] Goldstein BA, Polley EC, Briggs FB (2011). Random forests for genetic association studies. Stat Appl Genet Mol Biol.

[22] Cherkassky V (1997). The nature of statistical learning theory∼. IEEE Trans Neural Netw.

[23] Xie S, Ogden RT (2024). Functional support vector machine. Biostatistics.

[24] Yanagawa R, Iwadoh K, Akabane M, Imaoka Y, Bozhilov KK, Melcher ML, Sasaki K (2024). LightGBM outperforms other machine learning techniques in predicting graft failure after liver transplantation: Creation of a predictive model through large-scale analysis. Clin Transplant.

[25] Esteva A, Robicquet A, Ramsundar B, Kuleshov V, DePristo M, Chou K, Cui C (2019). A guide to deep learning in healthcare. Nat Med.

[26] Choi E, Schuetz A, Stewart WF, Sun J (2017). Using recurrent neural network models for early detection of heart failure onset. J Am Med Inform Assoc.

[27] Jensen PB, Jensen LJ, Brunak S (2012). Mining electronic health records: towards better research applications and clinical care. Nat Rev Genet.

[28] Adler-Milstein J, Jha AK (2017). HITECH act drove large gains in hospital electronic health record adoption. Health Aff (Millwood).

[29] Litjens G, Kooi T, Bejnordi BE, Setio AAA, Ciompi F, Ghafoorian M, van der Laak J (2017). A survey on deep learning in medical image analysis. Med Image Anal.

[30] Saeys Y, Inza I, Larranaga P (2007). A review of feature selection techniques in bioinformatics. Bioinformatics.

[31] Van Calster B, Nieboer D, Vergouwe Y, De Cock B, Pencina MJ, Steyerberg EW (2016). A calibration hierarchy for risk models was defined: from utopia to empirical data. J Clin Epidemiol.

[32] Steyerberg EW, Harrell FE (2016). Prediction models need appropriate internal, internal-external, and external validation. J Clin Epidemiol.

[33] Altman DG, Vergouwe Y, Royston P, Moons KG (2009). Prognosis and prognostic research: validating a prognostic model. BMJ.

[34] Collins GS, Reitsma JB, Altman DG, Moons KG (2015). Transparent reporting of a multivariable prediction model for individual prognosis or diagnosis (TRIPOD): the TRIPOD statement. BMJ.

[35] Shortliffe EH, Sepulveda MJ (2018). Clinical decision support in the era of artificial intelligence. JAMA.

[36] Obermeyer Z, Emanuel EJ (2016). Predicting the future - big data, machine learning, and clinical medicine. N Engl J Med.

[37] Sengupta PP, Huang YM, Bansal M, Ashrafi A, Fisher M, Shameer K, Gall W (2016). Cognitive machine-learning algorithm for cardiac imaging: a pilot study for differentiating constrictive pericarditis from restrictive cardiomyopathy. Circ Cardiovasc Imaging.

[38] Churpek MM, Yuen TC, Winslow C, Meltzer DO, Kattan MW, Edelson DP (2016). Multicenter comparison of machine learning methods and conventional regression for predicting clinical deterioration on the wards. Crit Care Med.

[39] Bardakjian T, Gonzalez-Alegre P (2018). Towards precision medicine. Handb Clin Neurol.

[40] Ginsburg GS, Phillips KA (2018). Precision medicine: from science to value. Health Aff (Millwood).

[41] Krumholz HM (2014). Big data and new knowledge in medicine: the thinking, training, and tools needed for a learning health system. Health Aff (Millwood).

[42] Van den Broeck J, Cunningham SA, Eeckels R, Herbst K (2005). Data cleaning: detecting, diagnosing, and editing data abnormalities. PLoS Med.

[43] Kahn MG, Raebel MA, Glanz JM, Riedlinger K, Steiner JF A pragmatic framework for single-site and multisite data quality assessment in electronic health record-based clinical research. Med Care. 2012;50.

[44] Rudin C (2019). Stop explaining black box machine learning models for high stakes decisions and use interpretable models instead. Nat Mach Intell.

[45] Debray TP, Vergouwe Y, Koffijberg H, Nieboer D, Steyerberg EW, Moons KG (2015). A new framework to enhance the interpretation of external validation studies of clinical prediction models. J Clin Epidemiol.

[46] Badhib A, Alshehri S, Cherif A (2025). IoT Authentication in Federated Learning: Methods, Challenges, and Future Directions. Sensors (Basel).

[47] Holzinger A, Langs G, Denk H, Zatloukal K, Muller H (2019). Causability and explainability of artificial intelligence in medicine. Wiley Interdiscip Rev Data Min Knowl Discov.

[48] Huang SC, Pareek A, Seyyedi S, Banerjee I, Lungren MP (2020). Fusion of medical imaging and electronic health records using deep learning: a systematic review and implementation guidelines. NPJ Digit Med.

[49] Rieke N, Hancox J, Li W, Milletari F, Roth HR, Albarqouni S, Bakas S (2020). The future of digital health with federated learning. NPJ Digit Med.

[50] Topol EJ (2019). High-performance medicine: the convergence of human and artificial intelligence. Nat Med.

[51] Jiang F, Jiang Y, Zhi H, Dong Y, Li H, Ma S, Wang Y (2017). Artificial intelligence in healthcare: past, present and future. Stroke Vasc Neurol.

